# Study of TiO_2_ and Al_2_O_3_ Nanoparticles’ Influence on the Variatropic Concrete Properties

**DOI:** 10.3390/ma19061081

**Published:** 2026-03-11

**Authors:** Evgenii M. Shcherban’, Sergey A. Stel’makh, Alexey N. Beskopylny, Levon R. Mailyan, Diana M. Shakhalieva, Andrei Chernil’nik, Vakhtang P. Matua, Denis A. Nikolenko

**Affiliations:** 1 Department of Engineering Geometry and Computer Graphics, Don State Technical University, 344003 Rostov-on-Don, Russia; au-geen@mail.ru; 2 Department of Unique Buildings and Construction Engineering, Don State Technical University, 344003 Rostov-on-Don, Russia; sergej.stelmax@mail.ru (S.A.S.); lrm@aaanet.ru (L.R.M.); chernila_a@mail.ru (A.C.); 3 Department of Transport Systems, Faculty of Roads and Transport Systems, Don State Technical University, 344003 Rostov-on-Don, Russia; 4 Department of Design, Don State Technical University, 344003 Rostov-on-Don, Russia; diana.elshaeva@yandex.ru; 5 Department of Highways, Faculty of Roads and Transport Systems, Don State Technical University, 344003 Rostov-on-Don, Russia; vmatua@donstu.ru (V.P.M.); d.a.nikolenko@mail.ru (D.A.N.)

**Keywords:** aluminum oxide nanoparticles, titanium oxide nanoparticles, modification, structure, compressive strength, frost resistance, variatropic concrete

## Abstract

Currently, one of the major trends in the construction industry is the creation of structures with increased strength and durability. The solution is the use of nanomaterials as modifiers for cementitious composites. The aim of this study is to produce concretes with a variable structure modified with a combination of aluminum oxide (NA) and titanium oxide (NT) nanoparticles with improved properties. A variatropic structure is characterized by differences in properties across the cross-section of the material. Concretes were produced using vibration (V), centrifugation (C), and vibrocentrifugation (VC) technologies. Modification was carried out with NA particles from 0% to 4.0% in increments of 1.0% and NT from 0% to 2.0% in increments of 0.5% of the binder mass. Through experimental study, the impact of combined nanomodification on the compressive strength, water absorption, and frost resistance of concrete created with different technologies was investigated. The most effective modification dosages with NA and NT particles were determined to be 2% and 1%. The determination of concrete properties and the statistical processing of experimental results were carried out in accordance with the requirements of standardized methods. Compared to control samples, the maximum compressive strengths for V, C, and VC concretes were 12.4%, 17.5%, and 20.3% higher, reaching 48.9 MPa, 58.4 MPa, and 62.9 MPa, respectively. The lowest water absorptions for V, C, and VC concretes were 5.21%, 4.24%, and 3.76%, which are 18.5%, 24.4%, and 29.2% lower than those of the control samples. After a series of freeze–thaw cycles—6 for V, 8 for C, and 10 for VC—the losses in compressive strength and mass of the nanomodified composites were less than those of the control samples, indicating an increase in the frost resistance of concrete. The influence of concrete production technology on the effect of nanomodification with NA and NT particles was proven. Nanomodified C and VC concretes have improved physical and mechanical properties compared to V concretes. Nanomodified concretes with a variable structure have a more organized microstructure with a greater number of clusters of calcium silicate hydroxides. The resulting variable-structure concrete has improved properties and can be used to manufacture columns, piles, and transmission line supports.

## 1. Introduction

In the modern construction industry, there is a high demand for structural elements with increased strength and durability. Concrete is a key and most popular building material worldwide, and its consumption is growing every year [1,2,3]. The most popular way to improve the properties of concrete is to enhance it with various types of additives. Among the vast variety of additives, nanomaterials have unique physicochemical properties and surface effects that can significantly affect the properties of concrete [4,5,6]. Silicon dioxide nanoparticles (NS), carbon nanotubes (CNTs), and calcium carbonate nanoparticles (NC) are most often used for concrete modification [7,8]. For example, modifying marine concrete with 2% NS improves its strength properties by up to 15% and reduces the carbonation depth by up to 20.5% [9]. Treating recycled aggregate with NS particles when adding it to concrete increases the strength properties of the composite [10]. The addition of NS can effectively increase the heat resistance of foam concrete and improve its thermal stability [11]. Modification of reinforced concrete with 2% NS improves frost resistance and corrosion resistance of concrete [12]. NS reduces the number of pores in the composite structure and increases its compressive strength and durability [13,14,15,16]. The inclusion of carbon nanotubes in the cement paste matrix helps to reduce the number of microcracks and improves its wear resistance. According to research [17], increasing the CNTs to 0.2% can boost the composite’s strength by as much as 42.45%. The mechanical properties of the ultra-high-strength concrete mixture are significantly improved by vibrational mixing and the introduction of CNTs [18]. Concrete composites modified with CNTs have an improved matrix with strong interphase adhesion and higher mechanical properties [19,20,21,22]. Calcium carbonate (NC) nanoparticles accelerate the hydration reactions of Portland cement due to nucleation and chemical effects [23]. NC particles in an amount of 2%, introduced into the composition of environmentally friendly self-compacting concrete with cotton fibers and recycled glass, contribute to the formation of a denser composite structure and improve its mechanical properties [24]. Including 1.5% NC increases the compressive and tensile strength by up to 37% and increases the impact resistance of concrete [25]. NC can improve the corrosion resistance of concrete used in soils with a high sulfate content [26,27]. Increases in the strength properties and durability of various types of concrete due to their modification with NC particles are also confirmed by other studies [28,29,30].

Among the many types of nanomaterials, aluminum oxide (NA) and titanium oxide (NT) nanoparticles attract special attention. NA and NT particles have good chemical compatibility with Portland cement and improve the properties of cement composites [31]. For example, modification of concrete with 1% NA increases its strength, reduces shrinkage, and improves the structure [32,33]. When included in the composition of cellular concrete, NA acts as a stabilizer and significantly increases their strength properties [34]. After 150 freeze–thaw cycles, the concrete made with recycled aggregate and modified NT exhibited greater capillary water absorption and a less dense structure than the modified NT concrete containing recycled aggregate, as reported in reference [35]. Concrete with 2% NT has improved frost resistance and lower porosity [36]. Concrete with NT particles has increased sulfate resistance [37]. The effectiveness of nanomodification of cement composites with NA, NT and their combinations, determined by the increase in mechanical properties, improvement of microstructure, and durability properties, is also confirmed by the works of other authors [38,39,40]. Based on the results of the literature review, we note that the assessment of the effectiveness of the use of nanomaterials has been mainly studied on various types of composites (heavy concrete, cellular concrete, self-compacting and ultra-high-strength concrete) manufactured using the classical technology of compaction by vibration effects [41,42,43]. It is worth noting that when modifying concrete simultaneously with several types of nanoparticles, there are limitations and problems associated with the specific interaction of key concrete components and the complexity of technologies for dispersing nanoadditives and introducing them into concrete. There is a clear shortage of studies devoted to an integrated approach, including the assessment of the effect of nanomaterials and their combinations in cement composites manufactured using centrifugal compaction technologies—centrifugation and vibrocentrifugation. A key characteristic of centrifugation and vibrocentrifugation is their ability to produce a composite with a distinctive variatropic structure. During the compaction process, centrifugal forces act on the coarse aggregate grains and displace them closer to the outer layer of the formed concrete element of the ring cross-section. Smaller grains of coarse aggregate and the cement-sand mixture are predominantly concentrated in the middle layer of the concrete element. The inner layer comprises a mixture of fine sand and cement particles [44,45]. Additional vibration effects, created by clamps with projections on the machine’s shafts, distinguish vibrocentrifugation technology. The structure of vibrocentrifuged elements of the ring cross-section is distinguished by a more uniform distribution of coarse aggregate grains between the outer and middle layers [46]. During the centrifugation and vibrocentrifugation of the concrete mixture, water migration occurs. Under the action of centrifugal forces, water in the concrete mixture, as the least dense component, is squeezed toward the inner surface of the product [47]. Studying the properties of concretes produced using centrifugation and vibrocentrifugation technologies and modified with nanomaterials is poorly understood and is of high relevance [48,49]. The scientific novelty of the work lies in the development of new types of concretes produced using centrifugation and vibrocentrifugation technologies and modified with a combination of nano-Al_2_O_3_ (NA) and nano-TiO (NT) particles, and the identification of the mechanisms of action of nanoadditives in the structure of composites under various technological parameters of their manufacture.

The objective of this study is to produce concrete with a variable structure, modified with NA and NT nanoparticles, with improved performance for the construction of industrial and civil facilities.

The objectives of the study included:

– Determining the physical properties of raw materials (Portland cement, sand, and crushed stone) and their particle size distribution; analyzing the morphology of NA and NT particles to determine their elemental chemical composition;
– Producing experimental specimens of vibrated, centrifuged, and vibrocentrifuged concrete modified with a combination of NA and NT nanoadditives in varying dosages;
– Sawing experimental specimens of centrifuged and vibrocentrifuged concrete with annular cross-sections into 100 × 100 × 100 mm cube specimens;
– Determining the properties of experimental concrete specimens produced using various technologies with varying NA and NT dosages at 28 days of age: compressive strength, water absorption, and frost resistance;
– Study of the structural features of the developed composites using SEM methods;
– Analysis of experimental results, selection of the most effective combination of NA and NT, and interpretation of the mechanisms of nanomaterials’ operation in the structure of cement matrices produced by centrifugation and vibrocentrifugation methods.

## 2. Materials and Methods

### 2.1. Materials

The following raw materials were used to produce variable-density concrete.

1. Portland cement CEM I 52.5 N (PC) (CEMROS, Stary Oskol, Russia). Properties: Blaine specific surface area—280 m^2^/kg; initial setting time—180 min; final setting time—230 min; normal consistency—24%; compressive strength after 28 days—48.6 MPa; flexural strength after 28 days—6.2 MPa. Chemical composition: SiO_2_—21.34%; Al_2_O_3_—4.48%; Fe_2_O_3_—5.71%; CaO—63.18%; MgO—1.43%; SO_3_—2.46%; Cl—0.03%; Alkali oxides calculated as Na_2_O—0.42%; LOI—0.98%.
2. Quartz sand (QS) (Arkhipovsky quarry, Arkhipovskoye, Russia). Properties: bulk density—1348 kg/m^3^; apparent density—2575 kg/m^3^; the content of dust and clay particles—0.12%.
3. Crushed granite stone, fraction 5–20 mm (CrS) (Granite, Kamennogorskoye, Russia). Properties: bulk density—1450 kg/m^3^; apparent density—2652 kg/m^3^; resistance to fragmentation—11.2 wt %; the content of lamellar and acicular grains—9.5 wt %.
4. Plasticizer PK1 (P) (Polyplast, Moscow, Russia). Properties: density—1.11 g/cm^3^.
5. Nanosized aluminum oxide (NA) (Shandong Tiancheng Chemical Co., Ltd., Jining, China).
6. Nanosized titanium oxide (NT) (Shandong Tiancheng Chemical Co., Ltd., Jining, China).

**Figure 1:**
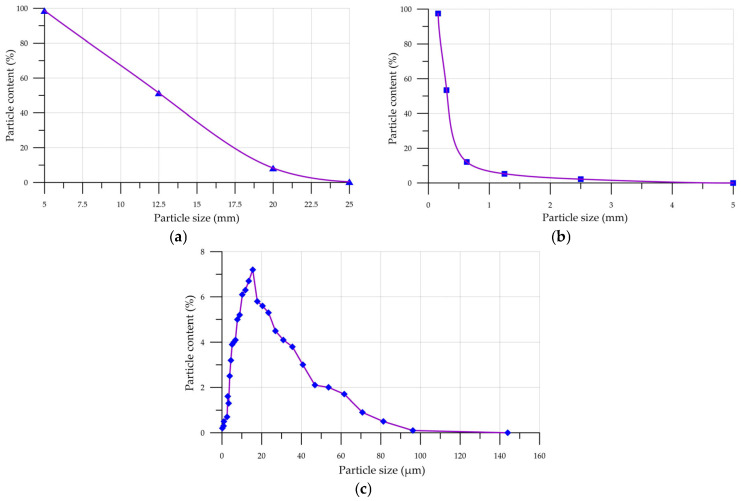
Particle size distribution curves: (**a**) QS; (**b**) CrS; (**c**) PC.

The particle size distribution curves of cement, sand, and crushed stone are shown in Figure 1.

According to Figure 1a, the total residuals for crushed stone on sieves with numbers 5, 12.5, 20, and 25 mm were 0.4%, 8.3%, 52.4%, and 98.7%, respectively. For sand (Figure 1b), the total residuals on sieves with numbers 5, 2.5, 1.25, 0.63, 0.315, and 0.16 were 0%, 2.2%, 5.3%, 12.1%, 53.4%, and 97.5%, respectively. The highest proportion of cement particles (Figure 1c)—80.8%—lies in the size range from 3 to 31 µm.

**Figure 2:**
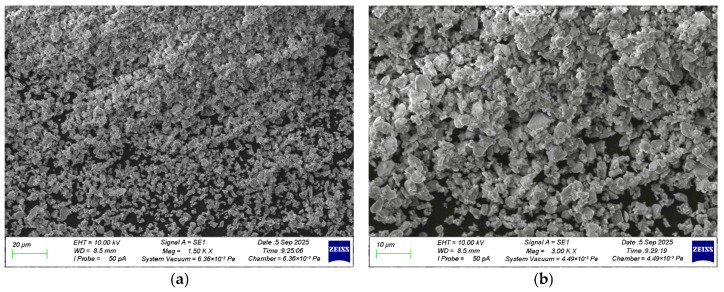
SEM image of NA: (**a**) at 1500× magnification; (**b**) at 3000× magnification.

**Figure 3:**
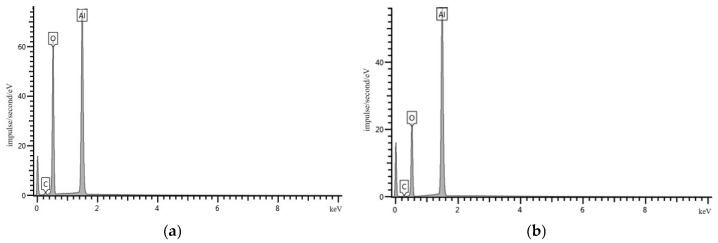
EDS of NA particles: (**a**) spectrum 1; (**b**) spectrum 2.

**Figure 4:**
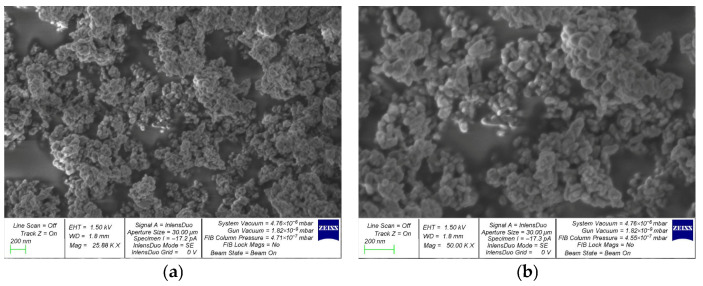
SEM image of NT: (**a**) at 25,880× magnification; (**b**) at 50,000× magnification.

**Figure 5:**
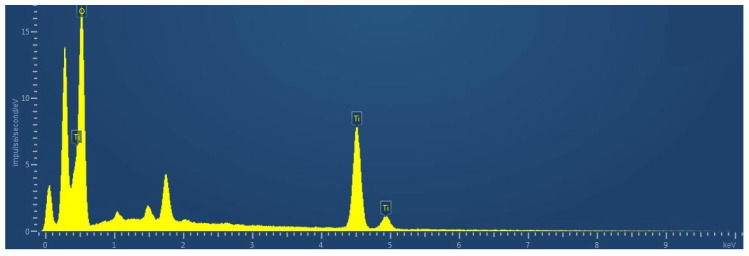
EDS of NT particles.

Figure 2, Figure 3, Figure 4 and Figure 5 show the results of SEM and EDS analyzes of NA and NT particles. Figure 2 shows photographs of the NA microstructure.

Analysis of the NA particle morphology revealed that these particles are agglomerated. Clusters of smaller particles are observed on the surface of larger ones. The NA particles are predominantly plate-like and angular in shape. Figure 3 shows the EDS of the NA particles.

According to the EDS results, NA particles contain the following chemical elements: Al, O, and C. Spectrum 1: O—58.19%; Al—38.65%; C—3.15%. Spectrum 2: O—43.31%; Al—54.93%; C—1.76%. Figure 4 shows SEM images of NT particles.

The average particle diameter (D50) is 20–30 nm. According to the manufacturer, the particles have a specific surface area of 130 m^2^/g.

Morphology analysis revealed that the NT particles are agglomerated. Clusters of smaller particles are observed on the surfaces of larger ones. The NT particles are predominantly spherical. Figure 5 shows the EDS of the NT particles.

According to the results of EDS analysis, NT particles have the following chemical elements: Ti—69.34%; O—30.66%.

The average particle diameter (D50) of NT is 30–50 nm. The specific surface area of NT particles is 50 m^2^/g. The crystal structure is rutile (manufacturer’s data).

The particle sizes of PC determined during particle size analysis primarily range from 3 to 31 µm and have a significant impact on the dispersion of nanoparticles in the cement matrix. NA and NT nanoparticles have high surface energy and actively attach to larger cement grains during hydration reactions, resulting in the formation of strong crystalline bonds.

### 2.2. Methods

During the development of experimental concrete compositions produced using vibration (V), centrifugation (C), and vibrocentrifugation (VC) technologies, studies by other authors on the nanomodification of concrete with NA and NT particles were additionally analyzed, and optimal modification ranges were determined [36,50]. In addition, preliminary experimental studies were conducted, modifying cement pastes with NA and NT particles separately and with a combination of both. Based on the determination of their properties, it was established that combined modification with both types of nanoparticles yields the best results, which determined the program for further research. The modification was carried out using a combination of NA and NT nanoparticles. Calculations for the NA particle quantity were performed using the cement mass, with increments of 1.0% covering a range of 0% to 4.0%. The amount of NT particles was calculated based on the cement mass in the range from 0% to 2.0% in 0.5% increments. Variatropic concretes modified with NA and NT particles were manufactured according to the formula presented in Table 1, observing the process specifications. Concrete mixes for the experimental samples were prepared using a standard method. First, all raw materials were dosed according to the formula presented in Table 1. The W/C value for all mixtures remained constant at 0.4. The raw materials were loaded into a laboratory concrete mixer in the following sequence: PC, QS, CrS, and mixing water with dispersed nanoparticles and a plasticizing additive. The mixture was then mixed until a homogeneous consistency was achieved. The finished concrete mix was removed from the mixer, and the samples were molded using the V, C, and VC technologies.

Mixing NA and NT particles with water and a plasticizing additive is an important process step and is performed to prevent possible agglomeration of nanoparticles and ensure their most uniform distribution within the structure of the future composite. The introduction of NA and NT particles into the mixing water was accomplished using ultrasonic irradiation using the following technology. Pre-measured quantities of nanoadditives and plasticizer were added to the water, after which a dispersant was immersed in the water and ultrasonicated for 8 min. After irradiation, the solution was visually assessed, checking for uniform particle distribution throughout the volume and the presence of nanoparticle agglomerates.

1. *Vibration Technology*. The concrete mix was loaded into 100 × 100 × 100 mm^3^ cube molds in three stages. The molds containing the mix were compacted on a laboratory vibrating platform for 60 s. The surface of the samples was then smoothed, the samples were aged for 24 h, and then removed from the molds. The vibrated concrete samples were cured under natural conditions for 28 days.

2. *Centrifugation and vibration centrifugation technologies.* The production of C and VC samples was carried out on a specialized laboratory setup [51]. The concrete mix was loaded into centrifugation molds, and the sample molding process was carried out with the following centrifugation parameters: concrete mix distribution speed over the metal mold—from 50 to 100 rpm; distribution time—6 min; intensive concrete mix compaction speed—600 rpm; intensive compaction time—8 min; total cycle duration—14 min. Vibratory centrifugation was carried out with similar parameters, and special clamps with 5 mm high, 20 mm long, and 30 mm pitch protrusions were placed on the unit shafts to create vibrations. After the molding process was complete, the slurry was drained from the molds, and the specimens were kept in the molds for 24 h before being removed. The centrifuged and vibrocentrifuged concrete specimens were cured under natural conditions for 28 days. After 28 days of curing, the annular specimens, with an outer diameter of 450 mm, an inner diameter of 150 mm, and a height of 1200 mm, were cut on a stone-cutting machine into cubes measuring 100 × 100 × 100 mm^3^, according to the scheme described in the study [52].

**Figure 6:**
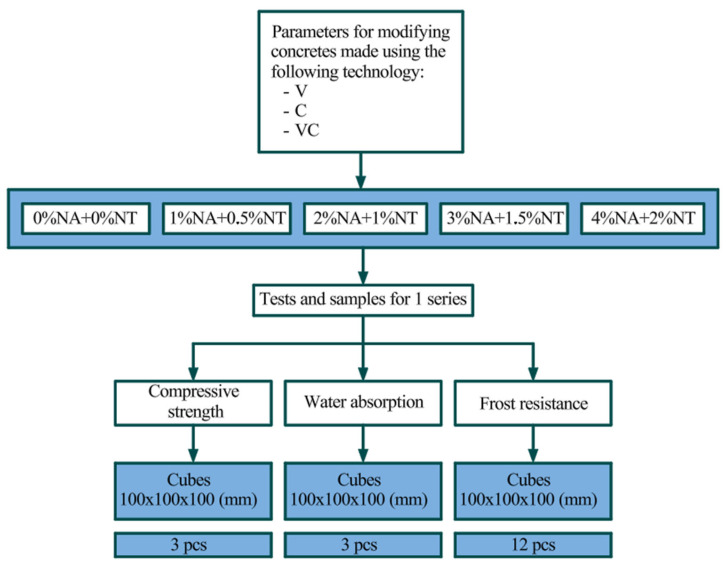
Experimental Research Layout.

The scheme of experimental studies of vibrated and variable-temperature concrete modified with a combination of nanoparticles is presented in Figure 6.

Experimental concrete specimens were tested for compressive strength, water absorption, and frost resistance. Forty-five cube specimens were used for strength testing, 45 for water absorption, and 180 for frost resistance, for a total of 270 cube specimens. All laboratory tests were conducted in accordance with standardized methodologies.

1. Compressive strength of V, C, and VC concrete (R, MPa) [53,54,55,56]. Cube specimens were installed in a testing machine and loaded to failure at a constant rate of (0.6 ± 0.2) MPa/s. Compressive strength was calculated using Formula (1) with an accuracy of 0.1 MPa. The compressive strength of a series of specimens was calculated as the arithmetic mean of three specimens.

(1) $$R=\alpha \frac{F}{A}$$

Here *F* is the ultimate load (N);

*A* is the cross-sectional area of the specimen (mm^2^);

*α* is a coefficient accounting for the specimen sizes (for specimens with a side of 100 mm, α = 0.95).

2. Water absorption of V, C, and VC concrete (W, %) [57]. Experimental concrete specimens were placed in a container filled with water such that the water level in the container was 50 mm above the specimen level. The water temperature in the containers was (20 ± 2) °C. The specimens were weighed every 24 h of water absorption until the results of two consecutive weighing differed by no more than 0.1%. Water absorption was calculated using Formula (2) with an accuracy of 0.1%. The water absorption of a series of specimens was calculated as the arithmetic mean of three specimens.

(2) $$W=\frac{m_{w}-m_{d}}{m_{d}}\;\times \;100$$

Here $m_{w}$ is the mass of the water-saturated specimen (g);

$m_{d}$ is the mass of the dried specimen (g).

3. *Frost resistance of V, C, and VC concrete* [58]. The base and control concrete specimens were saturated with a 5% aqueous sodium chloride solution before testing. The water-saturated control specimens were then tested for compressive strength. The base specimens were placed in a freezer in closed containers filled with a 5% aqueous sodium chloride solution so that the distance between the container walls, as well as between the container walls and the chamber, was at least 50 mm. After loading the chamber, the temperature was lowered for 2–3 h until the sodium chloride solution reached a temperature of −10 °C. The temperature was then lowered over 2.5 ± 0.5 h until the temperature in the aqueous sodium chloride solution reached minus 50 °C to minus 55 °C and maintained for 2.5 ± 0.5 h. The temperature was then raised to minus 10 °C over 1.5 ± 0.5 h, after which 100 × 100 × 100 mm^3^ specimens (in containers with lids) were thawed in a 5% aqueous sodium chloride solution at 20 ± 2 °C for 2.5 h. After the specified number of cycles, the control specimens were inspected, wiped with a damp cloth, weighed, and their compressive strength was determined. The strength results for the control and control specimens were processed using Formulas (3)–(7) (Table 2).

The specimens were considered having passed the frost resistance test if the ratio $X_{\min}^{II}\geq 0.9X_{\min}^{I}$ was observed, the mass loss did not exceed 2%, and the specimens were free of cracks, chips, or flaking of the edges. The above-described method for determining frost resistance pertains to the third accelerated method, where the climatic chamber ensures that the freezing temperature of an aqueous solution of sodium chloride is achieved and maintained from –50 °C to –55 °C. According to [58], frost resistance grade F_1_200 is assigned if concrete specimens withstand 5 to 7 freeze–thaw cycles and the ratio $X_{min}^{ll}\;\geq \;{0.9X}_{min}^{I}$ is observed, mass loss does not exceed 2% and there are no defects. Frost resistance grade F1300 is assigned if concrete specimens withstand 8 to 11 freeze–thaw cycles.

4. The morphology of nanosized aluminum oxide and titanium oxide particles, their chemical composition, and the microstructural features of the modified variatropic composites were assessed using scanning electron microscopy using a ZEISS CrossBeam 340 microscope—a dual-beam scanning electron-ion microscope equipped with an Oxford Instruments X-Max 80 X-ray microanalyzer (Carl Zeiss Microscopy GmbH, Jena, Germany).

## 3. Results and Discussion

**Figure 7:**
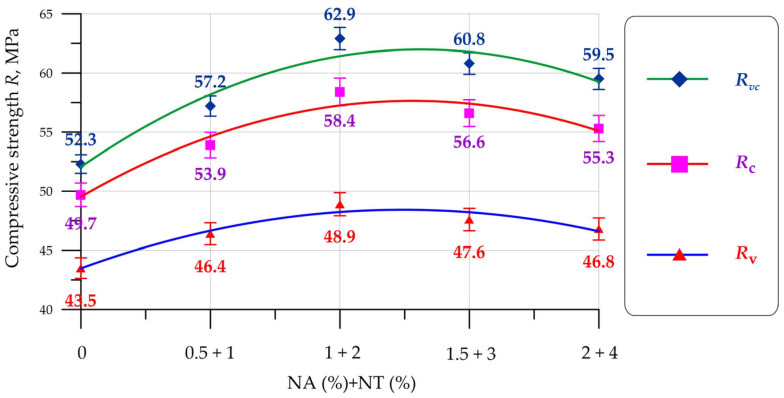
Compressive strength of V (*R*_v_), C (*R*_c_), and VC (*R*_vc_) concretes versus NA and NT content.

Figure 7 shows the results for the compressive strength of vibrated (V), centrifuged (C), and vibrocentrifuged concrete (VC) that has been modified with NA and NT nanoparticles.

According to the data presented in Figure 7, modification of concretes produced using three different technologies with NA and NT nanoparticles has an exclusively positive effect on compressive strength. The dependence of compressive strength change for V, C, and VC concretes is identical. The maximum compressive strength values were recorded at NA and NT content of 2% and 1% of the binder weight. The greatest strength increase was 12.4% for vibrated concrete, 17.5% for centrifuged concrete, and 20.3% for vibrocentrifuged concrete. When including a combination of NA and NT nanoadditives in dosages of 1.5% + 3% and 2% + 4%, the modification effect decreases, which is evident from the increases in compressive strength presented in Table 3. When included in concrete, Al_2_O_3_ nanoparticles have high reactivity and enter hydration reactions at the initial stage, which ensures the formation of a greater amount of calcium silicate hydroxide gel (CSH). Al_2_O_3_ nanoparticles have a high specific surface area and actively interact with calcium hydroxide (Ca(OH)_2_), thereby reducing its amount in the cement matrix and filling voids and pores. Also, the pozzolanic activity of Al_2_O_3_ nanoparticles contributes to the improvement of strength properties at later stages of composite hardening [59]. The behavior of TiO_2_ nanoparticles when included in the cement matrix is similar to the behavior of Al_2_O_3_ nanoparticles. TiO_2_ particles uniformly distributed throughout the cement matrix possess a large specific surface area and energy and accelerate the formation of CSH gel. TiO_2_ nanoparticles act as a filler, being incorporated into the matrix structure, reducing the overall number of capillary pores [60]. The combination of two types of nanoadditives, NA and NT, provides a comprehensive, enhanced effect of the above-described mechanisms of nanoparticle action within the cement matrix structure. The reduction in the nanomodifying effect with NA content greater than 2% and NT content greater than 1% may be due to nanoparticle agglomeration and their uneven distribution within the matrix structure.

An analysis of the compressive strength gains of concrete produced using various V, C, and VC technologies revealed that, with identical combinations and dosages of NA and NT particles, centrifuged concrete exhibits higher gains than vibrated concrete. Vibrated concrete exhibits the greatest gains compared to both vibrated and centrifuged concrete. Thus, the influence of manufacturing technology on the effectiveness of nanomodifying additives has been demonstrated. Higher compressive strength gains are directly related to the mechanism of centrifugal compaction of the concrete mix. Centrifugal forces during compaction act on the concrete mix and all its components—the cement-sand mixture and the coarse aggregate separately—ensuring their migration throughout the entire thickness of the formed concrete ring-section element. Due to centrifugal action, NA and NT nanoparticles are more uniformly distributed within the composite matrix. The addition of vibration during centrifugal compaction further improves the uniformity of particle distribution within the composite [61].

**Figure 8:**
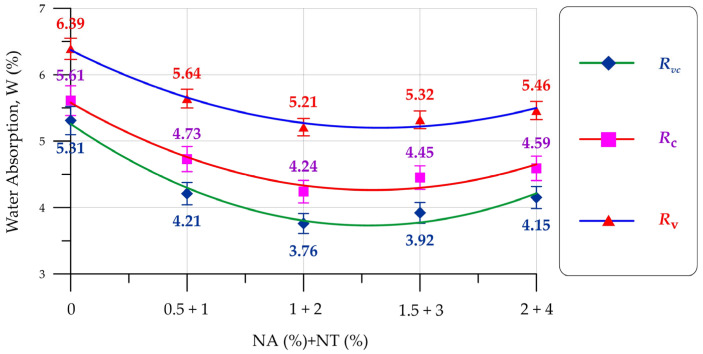
Water absorption of V, C, and VC concretes as a function of NA and NT content.

The results of determining the water absorption of V, C, and VC concretes modified with a combination of NA and NT nanoparticles are presented in Figure 8.

It was found that modifying V, C, and VC concretes with a combination of NA and NT nanoparticles in the ranges examined reduces their water absorption. The lowest water absorption values were recorded with 2% NA and 1% NT content. Compared to the control values, water absorption for V, C, and VC concretes decreased by 18.5%, 24.4%, and 29.2%, respectively. Al_2_O_3_ and TiO_3_ nanoparticles act as additional crystallization centers, forming dense clusters of CSH gel and a dense cement matrix around them. Al_2_O_3_ and TiO_3_ particles also fill voids and reduce the overall volume of capillary pores, resulting in a more organized and compact composite structure [62]. As with compressive strength, the influence of manufacturing technology on the overall effectiveness of the nanomodification has been demonstrated. This is enhanced by the effect of centrifugal force and vibration on the concrete mix during compaction. The quantitative reduction in water absorption depending on the NA and NT dosages and the concrete manufacturing technology is presented in Table 4.

**Figure 9:**
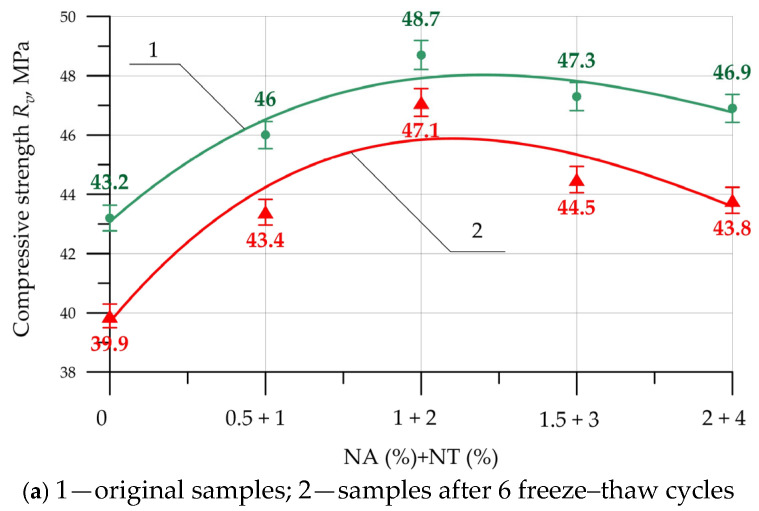

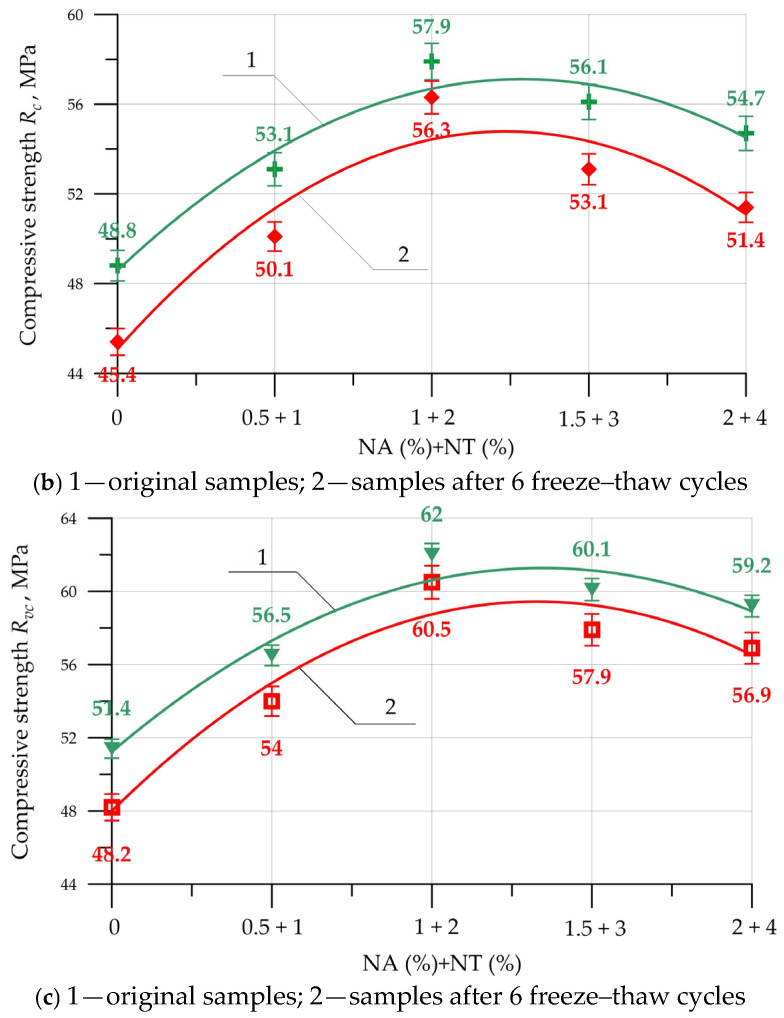
Compressive strength of concrete modified with NA and NT before and after freeze–thaw cycles: (**a**) V; (**b**) C; (**c**) VC.

The results of frost resistance of V, C, and VC concrete modified with NA and NT nanoparticles are presented in Table 5 and Figure 9.

Figure 9 shows the changes in compressive strength of V, C and VC concretes modified with a combination of NA and NT nanoparticles both before and after freezing-thawing.

Based on the analysis of the freeze–thaw resistance, the following was established for each concrete type:

– *Vibrated concrete*. Based on the data presented in Figure 9a, the maximum compressive strength was demonstrated by specimens modified with a combination of 2% NA and 1% NT. After six freeze–thaw cycles, the strength reduction for the base specimens ranged from a minimum of 3.3% for the V-2NA-1NT composition to a maximum of 7.6% for the control specimens. The mass of the main vibrated concrete samples of the following compositions after 6 freeze–thaw cycles decreased by 1.8%, 1.6%, 1.2%, 1.3%, and 1.4%, respectively, for the V-0NA-0NT, V-1NA-0.5NT, V-2NA-1NT, V-3NA-1.5NT, and V-4NA-2NT compositions. These percentages of mass reduction correlate well with their strength properties. The smallest mass loss of 1.2% was recorded for the V-2NA-1NT composition, which exhibits the highest strength. After 6 freeze–thaw cycles, the mass loss for all samples did not exceed 2%. Furthermore, based on the data presented in Table 5, the condition $X_{\min}^{II}\geq 0.9X_{\min}^{I}$ is satisfied for all V-series concretes modified with NA and NT. Accordingly, the samples are considered to have passed the frost resistance test and have a frost resistance grade of F200 in accordance with the requirements of regulatory documentation [58];
– *Centrifuged concrete*. Figure 9b shows that centrifuged concrete with 2% NA and 1% NT has a maximum compressive strength of 57.9 MPa. After 8 freeze–thaw cycles, the compressive strength for the main samples of the C-0NA-0NT, C-1NA-0.5NT, C-2NA-1NT, C-3NA-1.5NT, C-4NA-2NT compositions decreased by 7.0%, 5.6%, 2.8%, 5.3% and 6%, respectively, for the same compositions, the weight losses after 8 freeze–thaw cycles were 1.4%, 1.3%, 0.9%, 1.1% and 1.2%, respectively. The smallest decrease in compressive strength and weight loss were recorded for the C-2NA-1NT composition. After 8 freeze–thaw cycles, the weight loss for all samples did not exceed 2%. In addition, based on the data presented in Table 5, the condition $X_{\min}^{II}\geq 0.9X_{\min}^{I}$ is met for all series of C concretes modified with NA and NT, accordingly, the samples are considered to have passed the frost resistance test and have a frost resistance grade of F300 in accordance with the requirements of regulatory documentation [58];
– *Vibrocentrifuged concrete*. As in the case of V and C concretes, for VC concrete the maximum compressive strength of 62 MPa was recorded for the composition with 2% NA and 1% NT. After 10 freeze–thaw cycles, the decrease in compressive strength for the main specimens was 6.2%, 4.4%, 2.4%, 3.7%, and 3.9%, respectively; for the same compositions, the mass decrease after 10 freeze–thaw cycles was 1.0%, 0.8%, 0.7%, 0.9%, and 1.0%, respectively. After 10 freeze–thaw cycles, the mass loss for all specimens did not exceed 2%. Furthermore, based on the data presented in Table 5, the condition $X_{\min}^{II}\geq 0.9X_{\min}^{I}$ is satisfied for all series of VC concretes modified with NA and NT. Accordingly, the samples are considered having passed the frost resistance test and are assigned frost resistance grade F300 in accordance with regulatory requirements [58].

Modification of V, C, and VC concretes with a combination of NA and NT nanoadditives increases their resistance to alternating freeze-thaw cycles. This is explained by the following processes occurring during the formation of the composite structure. NA particles actively participate in hydration reactions and form additional CSH gels, which reduces the number of voids, pores, and Ca(OH)_2_ crystals in the cement matrix. NT particles have a predominantly filling effect and contribute to a reduction in the total number of capillary pores, which positively impacts the structure of the cement matrix. Thus, the presence of nano-additives in the composition of concrete provides better protection of its internal structure against freezing and thawing cycles [63,64]. Based on the water absorption and frost resistance measurements for V, C, and VC concretes modified with a combination of NA and NT, a clear correlation between decreased water absorption and increased frost resistance is evident. Freeze–thaw cycles are accompanied by phase transitions of water located in the pores of the concrete matrix. These processes cause internal stress, deform, and destroy the composite structure. Nanomodified concretes have a denser structure with fewer pores and, as a result, better resist aggressive freezing and thawing.

Based on the results of experimental studies of V, C, and VC concretes modified with a combination of NA and NT, the following has been established:

– The inclusion of 2% NA and 1% NT allows for the production of V, C, and VC concretes with maximum compressive strength values of 48.9 MPa, 58.4 MPa, and 62.9 MPa, respectively.
– The inclusion of 2% NA and 1% NT allows for the production of V, C, and VC concretes with minimum water absorption of 5.21%, 4.24%, and 3.76%, respectively.
– Modification of V, C, and VC concretes with NA and NT nanoadditives improves the frost resistance of the concretes. After 6 freeze–thaw cycles for V, 8 for C, and 10 for VC concrete, the compressive strength and mass losses of the nanomodified composites were lower than those of the control compositions of the V-0NA-0NT, C-0NA-0NT, and VC-0NA-0NT types.
– The influence of concrete manufacturing technology on the effect of nanomodification with NA and NT particles has been proven; centrifugal forces during centrifugation and centrifugal forces combined with vibration during vibrocentrifugation affect the concrete mixture during its compaction, ensure the migration of raw components across the entire wall thickness of the formed annular cross-section element, and promote a uniform distribution of nanoparticles in the cement matrix. Due to this, the formation of CSH gel around NA and NT nanoparticles occurs more uniformly throughout the entire structure of the composite and pores and voids are uniformly filled.

**Figure 10:**
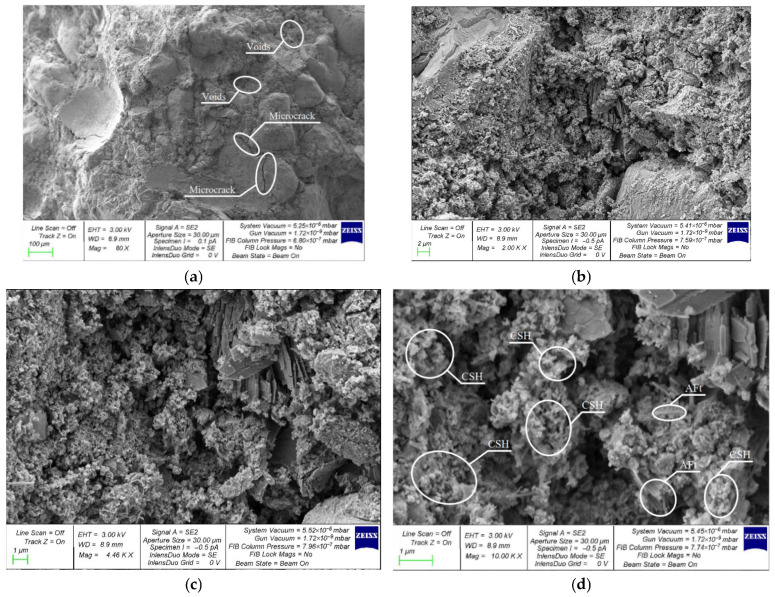
SEM of the V-2NA-1NT composite structure: (**a**) 80×; (**b**) 2000×; (**c**) 4460×; (**d**) 10,000×.

**Figure 11:**
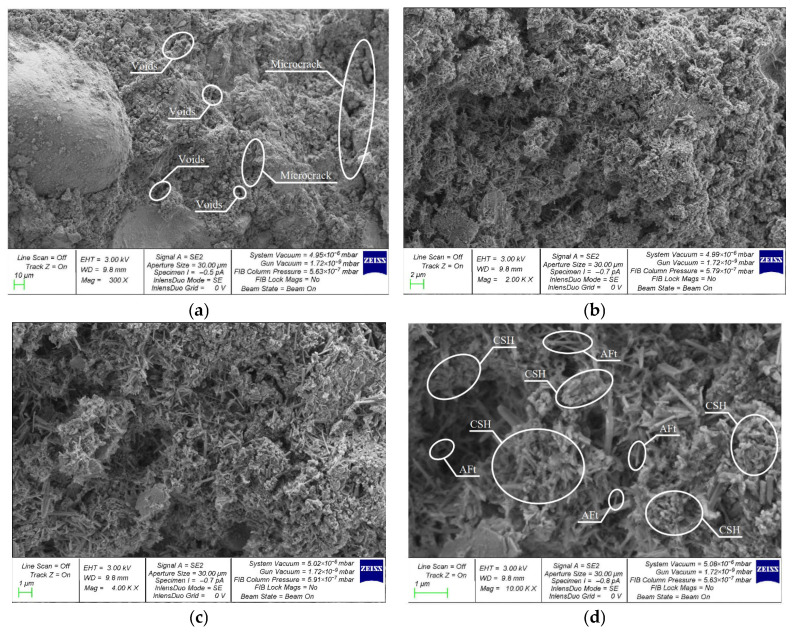
SEM structure of V-2NA-1NT: (**a**) 300×; (**b**) 2000×; (**c**) 4000×; (**d**) 10,000×.

**Figure 12:**
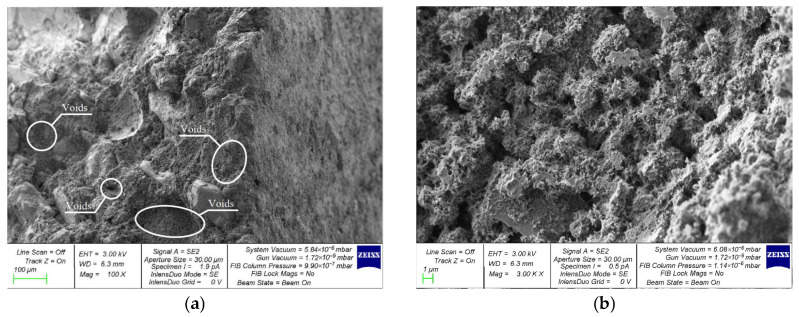

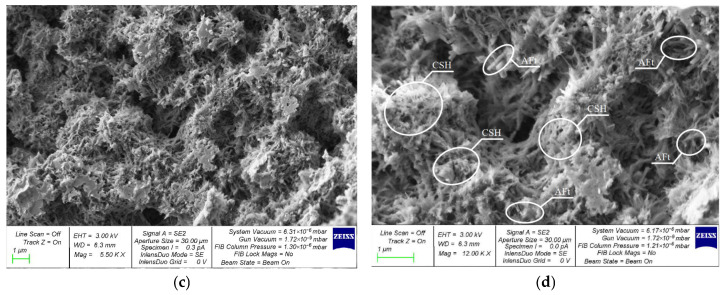
SEM structure of VC-2NA-1NT: (**a**) 100×; (**b**) 3000×; (**c**) 5500×; (**d**) 12,000×.

A comparative assessment of the microstructure of V, C and VC concretes modified with the best combination of 2% NA and 1% NT nanoparticles was performed on composite samples of the following compositions: V-2NA-1NT (Figure 10); C-2NA-1NT (Figure 11); and VC-2NA-1NT (Figure 12).

Figure 10 shows the microstructure of concrete produced using vibration technology and modified with the most effective combination of 2% NA and 1% NT particles. Micro-cracks and voids are visible in the composite structure. At higher magnifications, areas of CSH accumulation and ettringite crystals (AFt) are visible in the composite structure.

Nanomodified composites produced using centrifugation technology have a more uniform and well-organized structure compared to the structure of vibrated concrete. Microcracks and pores are also observed in the structure. CSH clusters are observed in larger numbers and are densely packed. Small amounts of (AFt) crystals are present in the spaces between the CSH clusters.

The structure of vibrocentrifuged concrete with NA and NT nanoparticles is more organized and ordered than that of vibrated and centrifuged concrete. The high density of CSH zones directly shows the superior strength properties of this composite.

Analysis of the research results identified the main mechanisms of complex nanomodification of V, C, and VC concrete: targeted formation of the composite structure at the nano- and micro-levels; accelerated and more complete hydration of Portland cement minerals; formation of additional CSH gels; filling of pores and voids; improvement of contact zones between the composite components.

The results obtained in this study for evaluating the strength properties and durability of composites modified with NA and NT nanoadditives, manufactured using three different technologies, are consistent and supported by the work of other authors (Table 6).

**Figure 13:**
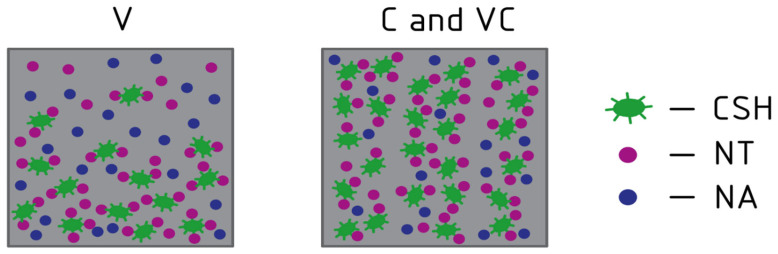
Schematic visualization of the distribution of NA and NT particles in the structure of cement matrices manufactured using V, C, and VC technologies.

A comparative analysis revealed that the modification ranges of NA and NT composites range from 0.5% to 4% inclusive. Relatively low dosages of these nanoadditives and their combinations provide significant improvements in the strength properties and durability of concrete, while microstructural analysis results demonstrate an improved and well-organized structure. A description of the specific operating mechanisms of the combination of NA and NT nanoadditives during the production of concrete using V, C, and VC technologies is of interest. Experiments have shown that when concrete is modified with the most effective combination of 2% NA and 1% NT nanoadditives, centrifuged and vibrocentrifuged concrete exhibit higher increases in compressive strength, lower water absorption values, and increased resistance to alternating freeze–thaw cycles. As noted above, the higher efficiency achieved with the inclusion of NA and NT nanoadditives is related to the mechanism of centrifugal compaction of the concrete mix, the effect of centrifugal force on the concrete mix, and the migration and distribution of concrete mix components across the annular cross-section. A schematic visualization of the distribution of NA and NT particles in the structure of cement matrices produced using vibration, centrifugation, and vibrocentrifugation technologies is shown in Figure 13. Due to the action of centrifugal forces, NA and NT particles migrate across the entire thickness of the annular cross-section and are ordered along the direction of the forces. Additional vibration effects during centrifugation contribute to a more uniform distribution of nanoparticles in the transverse direction.

This study has important scientific and practical implications for the construction industry. Modifying heavy-duty concrete with NA and NT nanomaterials in optimal quantities significantly improves their strength properties, reduces water absorption, and increases frost resistance. Improving these properties increases the durability of concrete [70]. The influence of concrete production technology on the overall effectiveness of combined modification with nanoadditives has been demonstrated. Concretes manufactured using centrifugation and vibrocentrifugation technologies exhibit higher increases in compressive strength compared to vibrated composites with the same modification parameters. Improving the durability of concrete is essential for the sustainable development of the entire construction industry. The service life of concrete structures primarily determines its ability to resist external aggressive influences. Increased durability allows concrete structures to be used for the estimated service life without additional costs for repair and restoration work or replacement work due to premature wear due to corrosion [71]. Perhaps the main limitation of this work is the lack of research assessing the resistance of variatropic nanomodified concretes to chloride and sulfate attack, as well as the lack of water resistance values. Future work will evaluate the resistance of nanomodified variatropic composites to aggressive chemical influences under temperature fluctuations, and the changes in the composite structure after cycles of such influences will be analyzed using SEM.

## 4. Conclusions

Concretes with variable structure modified with a combination of NA and NT nanoparticles were obtained, and their strength and durability properties were determined. Hardened concretes produced using V, C, and VC technologies and modified with varying doses of NA and NT were evaluated for compressive strength, water absorption, and frost resistance.

(1) The compressive strength of V, C, and VC concretes with the inclusion of NA and NT nanoparticles tended to increase in the studied ranges. The optimal doses of NA and NT nanoparticles were 2% and 1%, respectively. The maximum increases in compressive strength with these nanomodification parameters for V, C, and VC concretes were 12.4%, 17.5%, and 20.3%, respectively. Nano-Al_2_O_3_ and nano-TiO_2_ particles act as additional crystallization centers, promote the formation of additional CSH, and accelerate hydration reactions, which improves the organization of the composite structure and increases its strength.

(2) The water absorption of V, C, and VC concretes with the inclusion of NA and NT nanoparticles in the studied ranges tends to decrease. The lowest water absorption values were observed for V, C, and VC concretes modified with a combination of NA and NT particles in amounts of 2% and 1%, respectively. Compared to the control values, the water absorption reductions for each concrete type were 18.5%, 24.4%, and 29.2%, respectively. NA and NT densify the structure of the cement matrix and reduce its water absorption through the following mechanisms: they fill the voids between cement grains, accelerate the hydration process, and improve the adhesion between the aggregate grains and the cement paste.

(3) The inclusion of NA and NT particles increases the resistance of V, C, and VC concretes to alternating freeze–thaw cycles. Vibrated concrete with 2% NA and 1% NT after 6 freeze–thaw cycles exhibited the lowest values of compressive strength loss and mass loss, which amounted to 3.3% and 1.2%, respectively. The reduction in strength and mass loss for centrifuged concrete after 8 freeze–thaw cycles and vibrocentrifuged concrete after 10 freeze–thaw cycles modified with a similar combination of nanoadditives were 2.8%/0.9% and 2.4%/0.7%, respectively. The frost resistance of composites modified with NA and NT nanoadditives is increased by changing the pore structure of the concrete; its capillary porosity is reduced and structured.

(4) SEM analysis of the structure of V, C, and VC concretes modified with a combination of 2% NA and 1% NT reveals some differences in the composite structures. Variatropic concrete has a more organized structure with a greater number of clusters of calcium silicate hydrous zones.

(5) The influence of concrete production technology on the effect of nanomodification with NA and NT particles has been demonstrated. Directed centrifugal forces and additional vibration promote uniform distribution of nanoparticles across the entire thickness of the annular section and enhance the nanomodification effect, which is determined by the following key principles. Due to their high specific surface area, NA and NT nanoparticles actively interact with cement particles, accelerate hydration processes, and promote the formation of homogeneous hydrosilicate gels. Nanoparticles act as very fine agents, filling pores in the interphase zone and compacting the microstructure. Thus, the integrated combination of vibrocentrifugation technology and modification with nanoadditives allows for the production of a composite with an improved structure and enhanced performance properties.

(6) Nanomodified centrifuged and vibrocentrifuged ring-section concretes with improved properties can be used in the construction industry for the manufacture of columns, piles, and transmission line supports.

(7) The results obtained during the study make a significant contribution to the development of building composite materials with a variatropic (non-uniform cross-section) structure with improved performance properties due to the introduction of nano-sized additives into their composition.

## Figures and Tables

**Table 1:** Compositions V, C, and VC with flow rates per 1 m3.

Serial Number	Mixture Type	PC (kg/m^3^)	QS (kg/m^3^)	CrS (kg/m^3^)	W (L)	NA		NT		P (kg/m^3^)
						(%)	(kg/m^3^)	(%)	(kg/m^3^)	
1	V-0NA-0NT	410	521	1176	160	0	0.0	0	0	1.23
2	V-1NA-0.5NT	410	521	1176	160	1	4.1	0.5	2.05	1.23
3	V-2NA-1NT	410	521	1176	160	2	8.2	1	4.1	1.23
4	V-3NA-1.5NT	410	521	1176	160	3	12.3	1.5	6.15	1.23
5	V-4NA-2NT	410	521	1176	160	4	16.4	2	8.2	1.23
6	C-0NA-0NT	410	521	1176	160	0	0.0	0	0	1.23
7	C-1NA-0.5NT	410	521	1176	160	1	4.1	0.5	2.05	1.23
8	C-2NA-1NT	410	521	1176	160	2	8.2	1	4.1	1.23
9	C-3NA-1.5NT	410	521	1176	160	3	12.3	1.5	6.15	1.23
10	C-4NA-2NT	410	521	1176	160	4	16.4	2	8.2	1.23
11	VC-0NA-0NT	410	521	1176	160	0	0.0	0	0	1.23
12	VC-1NA-0.5NT	410	521	1176	160	1	4.1	0.5	2.05	1.23
13	VC-2NA-1NT	410	521	1176	160	2	8.2	1	4.1	1.23
14	VC-3NA-1.5NT	410	521	1176	160	3	12.3	1.5	6.15	1.23
15	VC-4NA-2NT	410	521	1176	160	4	16.4	2	8.2	1.23

**Table 2:** Calculation formulas for determining frost resistance.

Indicator	Calculation	Description
Change in sample mass	$∆m\;=\;\frac{m\;-\;m_{1}}{m}\cdot 100$ (3)	*m* is the mass of the specimen before freezing and thawing (g); *m*_1_ is the mass of the specimen after freezing and thawing (g)
Mean compressive strength	$X_{cp}\;=\;\frac{\sum X_{i}}{n}$ (4)	*X*i is the strength of one specimen, MPa; *n* is the number of specimens
Standard deviation	$\sigma_{n}\;=\;\frac{W_{m}}{\alpha }$ (5)	*W*_m_ is the range of individual concrete strength values in the series, defined as the difference between the maximum and minimum individual strength values, MPa α = 2.5
Lower confidence interval for control samples	$X_{min}^{I}\;=\;X_{cp}^{I}\;-\;t_{\beta }\sigma_{n}^{I}$ (6)	*t*_β_ = 2.57
Lower confidence interval for the main samples	$X_{min}^{II}\;=\;X_{cp}^{II}\;-\;t_{\beta }\sigma_{n}^{II}$ (7)	

**Table 3:** Change in compressive strength of concrete modified with a combination of NA and NT.

Change in Compressive Strength	Content NA (%) + NT (%)				
	0	1 + 0.5	2 + 1	3 + 1.5	4 + 2
∆*R*_v_ (%)	0	6.7	12.4	9.4	7.6
∆*R*_c_ (%)	0	8.5	17.5	13.9	11.3
∆*R*_vc_ (%)	0	9.4	20.3	16.3	13.8

**Table 4:** Change in water absorption of concrete modified with a combination of NA and NT.

Change in Water Absorption	Content of NA (%) + NT (%)				
	0	1 + 0.5	2 + 1	3 + 1.5	4 + 2
∆W_v_ (%)	0	−11.7	−18.5	−16.7	−14.6
∆W_c_ (%)	0	−15.7	−24.4	−20.7	−18.2
∆W_vc_ (%)	0	−20.7	−29.2	−26.2	−21.8

**Table 5:** Calculation of frost resistance of V, C, and VC concrete modified with NA and NT nanoparticles.

Mixture Type	∆m (%)	$X_{min}^{I}$	$X_{min}^{II}$	$0.9X_{min}^{I}$	$X_{min}^{II}\geq 0.9X_{min}^{I}$
V-0NA-0NT	1.8	41.3	37.5	37.2	Check passed
V-1NA-0.5NT	1.6	43.4	41.7	39.1	Check passed
V-2NA-1NT	1.2	47.1	45.6	42.3	Check passed
V-3NA-1.5NT	1.3	44.6	42.5	40.2	Check passed
V-4NA-2NT	1.4	43.7	41.7	39.3	Check passed
C-0NA-0NT	1.4	46.8	42.8	42.2	Check passed
C-1NA-0.5NT	1.3	50.9	48.7	45.8	Check passed
C-2NA-1NT	0.9	56.5	55.0	50.8	Check passed
C-3NA-1.5NT	1.1	54.4	51.0	48.9	Check passed
C-4NA-2NT	1.2	51.7	49.2	46.5	Check passed
VC-0NA-0NT	1.0	49.8	46.7	44.8	Check passed
VC-1NA-0.5NT	0.8	54.8	51.9	49.3	Check passed
VC-2NA-1NT	0.7	60.5	58.9	54.4	Check passed
VC-3NA-1.5NT	0.9	58.0	56.7	52.2	Check passed
VC-4NA-2NT	1.0	56.9	55.9	51.2	Check passed

**Table 6:** Effect of nanoadditives and their combinations on the properties of cementitious composites.

Reference Number	Type of Nanoparticle	Optimal Dosage	Result Obtained
[38]	nano-Al_2_O_3_ + nano-TiO_2_	0.5% + 1%	The pore structure of the concrete has improved. Compressive, splitting, and flexural strengths increased by 42%, 34%, and 28%, respectively, compared to control samples.
[64]	nano-Al_2_O_3_/nano-TiO_2_/nano-SiO_2_	Up to 4%	Mechanical properties and resistance to chemical attack, carbonation, and chloride ion penetration have been improved.
[59]	nano-Al_2_O_3_	2%	Compressive strength has increased by 61%. Water absorption has decreased by 46%. A significant increase in electrical resistance has been recorded, indicating improved durability.
[65]	nano-Al_2_O_3_/nano-TiO_2_/nano-Fe_2_O_3_	Up to 3%	The workability of self-compacting concrete mixtures has improved. Strength properties have significantly increased, and the porosity of the composite structure has been reduced.
[66]	nano-TiO_2_	0.4%	Increased resistance to freeze–thaw cycles
[32]	nano-Al_2_O_3_	Up to 3%	Increase in compressive strength by up to 12%. The composite has a denser structure.
[67]	nano-TiO_2_	Up to 0.5%	Improved mechanical strength and frost resistance.
[68]		2%	Increase in strength properties and improved durability.
[69]	nano-Al_2_O_3_	1%	Improvement in strength properties by up to 20% and elastic modulus by up to 14%.

## Data Availability

The original contributions presented in this study are included in the article. Further inquiries can be directed to the corresponding author.

## Abbreviations

The following abbreviations are used in this manuscript:

NA	Aluminum oxide nanoparticles
NT	Titanium oxide nanoparticles
V	Vibration
C	Centrifugation
VC	Vibrocentrifugation
CNTs	Carbon nanotubes
